# How plants cope with heavy metals

**DOI:** 10.1186/1999-3110-55-35

**Published:** 2014-03-20

**Authors:** Katrin Viehweger

**Affiliations:** Radiotherapeutics Division, Helmholtz-Zentrum Dresden-Rossendorf eV; Institute of Radiopharmacy, P.O. Box 510119, D-01314 Dresden, Germany

**Keywords:** Heavy metals, Tolerance, Toxicity, Signaling, Sequestration, Chelation

## Abstract

Heavy metals are naturally occurring in the earth‘s crust but anthropogenic and industrial activities have led to drastic environmental pollutions in distinct areas. Plants are able to colonize such sites due to several mechanisms of heavy metal tolerance. Understanding of these pathways enables different fruitful approaches like phytoremediation and biofortification.

Therefore, this review addresses mechanisms of heavy metal tolerance and toxicity in plants possessing a sophisticated network for maintenance of metal homeostasis. Key elements of this are chelation and sequestration which result either in removal of toxic metal from sensitive sites or conduct essential metal to their specific cellular destination. This implies shared pathways which can result in toxic symptoms especially in an excess of metal. These overlaps go on with signal transduction pathways induced by heavy metals which include common elements of other signal cascades. Nevertheless, there are specific reactions some of them will be discussed with special focus on the cellular level.

**Electronic supplementary material:**

The online version of this article (doi:10.1186/1999-3110-55-35) contains supplementary material, which is available to authorized users.

## Review

### Introduction

**Figure 1:**
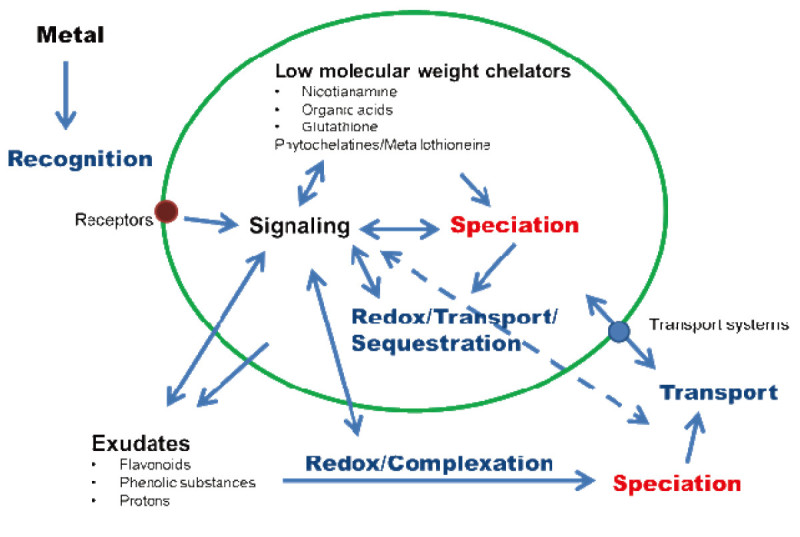
**Short overview about some important aspects of cellular metal interaction.** Arrows indicate interactions between different elements.

Basal heavy metal tolerance is presumably found in all plant species. Thereby, they run a complex system consisting of uptake/efflux, transport/sequestration and chelation (Figure 1). These key elements are involved tightly in homeostasis of essential metal micronutrients. The more or less characteristic of these elements divide the plant kingdom into two groups: (hyper)accumulating and non-accumulating plants.

This review will provide an overview about these tolerance mechanisms with focussing on the cellular level and signalling pathways induced by metals. The discussion of some examples will underline the multitude and complexity of signals and responses. It will trigger further work on responses towards heavy metals in plants especially in the way of low, environmentally relevant metal concentrations.

### Classification in non-and (hyper) accumulator plants

The majority of plants can be classified as non-accumulator plants. Nevertheless, all have to cope with heavy metals for nutrition purposes and growing in metalliferous soils, respectively. Hence, they have to possess finely tuned mechanisms for living with even toxic heavy metals (Hall 2002Clemens 2001,2006). The simplest strategy is to avoid metal uptake from soil or to exclude it preventing metal movement into shoots. Additionally, elements for the acquisition and sequestration of essential metals are often used. However, this can cause interferences with the plant metal homeostasis and probably induce toxic symptoms.

Such symptoms are manifold, for a review see (Fodor et al. 2002): They comprise impairments of chlorophyll synthesis resulting in chlorotic leaves, changed ratios of chlorophyll *a* and *b* (Murthy et al. 1984Viehweger and Geipel 2010Mysliwa-Kurdziel et al. 2004) and photosynthetic activity (Küpper et al. 2007), dwarfism of plants or effects on root ultrastructure (Barcelo et al. 2004) (Table 1). However, it is not clear if these impacts are either cause or consequence of metabolic perturbations in heavy metal exposed plants. Hence, it is necessary to investigate metal tolerance and toxicity on cellular and molecular level as it will be discussed in the following Sections.

**Table 1:** **Some examples of obvious toxic symptoms induced by metals**

Metal	Toxic symptoms	Reference
Excess or deficiency of copper, excess of uranium, zinc, cadmium	Impact on Photosynthetic apparatus: chlorotic leaves, changed ratios of chlorophyll *a* and *b*, decreasing net photosynthetic rate	Ouzounidou 1995Viehweger and Geipel 2010Monnet et al. 2001Prasad 1995reviewed in Mysliwa-Kurdziel et al. 2004
Excess of aluminium, cadmium, copper	Effects on root ultrastructure: inhibition of root elongation, increase in volume of cortex cells, damage to epidermis	Kidd et al. 2001Vazquez et al. 1992, Ouzounidou, 1995reviewed in Barcelo et al. 2004
Excess of aluminium, cadmium, lead	Lipid peroxidation of membranes – membrane leakage, change of lipid composition	Kochian 1995Hernandez and Cooke 1997Stefanov et al. 1995
Cadmium, lead, uranium	Changes in cellular concentrations of essential micronutrients like iron, calcium, manganese, zinc	Hernandez et al. 1998Zhang et al. 2000Viehweger and Geipel 2010

It should be noted that not only excessive metal concentrations causes toxicity.

A special case of metal tolerance is hypertolerance. Metal-hypertolerant plants except hyperaccumulators are able to exclude metals from their tissues in order to minimize metal accumulation especially in their aboveground tissues (Baker 1981). This is the key difference to hyperaccumulating plants. Nevertheless, metal hyperaccumulation is associated with metal hypertolerance revealing another strategy of detoxification.

Metal hyperaccumulating plants are characterized by a shoot/root ratio of metal accumulation > 1 (Baker et al. 1994). Such an outstanding metal accumulation is achieved by:

1. Overexpression of transport systems required for enhanced sequestration,
2. Tissue-specific expression of proteins,
3. High metal chelator concentrations.

Transcriptomic studies revealed that metal hyperaccumulation in *Arabidopsis halleri* has been associated with more than 30 candidate genes which are higher expressed compared with the nonaccumulator *A. thaliana* (Becher et al. 2004). (Pence et al. 2000) could show that the Zn^2+^ transporter ZnT1 is overexpressed in the hyperaccumulator *Noccaea caerulescens* compared to the non-accumulator *N. arvense*. Prominent examples are metal pumps belonging to the P_1B_-adenosine triphosphatase (heavy-metal ATPases, HMA) transporter family (Axelsen and Palmgren 1998). These transporters are able to transport different metals (like Zn^2+^, Cu^+^, Cu^2+^, Cd^2+^, Pb^2+^, Ni^2+^, Co^2+^) across biological membranes. Thereby, HMA2 and HMA4 drive metal efflux out of the cell in *A. thaliana* (Eren and Argüello 2004) and promote xylem loading of metal in *N. caeruslecens* (Papoyan and Kochian 2004). HMA4 is responsible for zinc hyperaccumulation in *A. halleri* as it was shown by a RNA interference approach for down-regulation of its expression. Additionally, transfer of the HMA4 gene to *A. thaliana* enables zinc partitioning into xylem vessels and up-regulated specific genes characteristic for zinc hyperaccumulators (Becher et al. 2004Hanikenne et al. 2008). This example shows impressively the importance of regulatory gene expression and gene copy number expansions for the special trait of metal hyperaccumulation. In contrast, HMA3 is localized at the tonoplast enabling vacuolar metal influx and therefore cellular sequestration (Gravot et al. 2004). Another vacuolar membrane transporter, the Zn/H^+^ antiporter (metal tolerance protein, MTP1 a member of cation diffusion facilitator (CDF) protein family) is highly overexpressed in the aforementioned hyperaccumulating plants compared to their closed related non-accumulators (Drager et al. 2004). MTP1 is able to create a metal sink in the shoots (Gustin et al. 2009) but there are controversial discussions about its importance for hyperaccumulation.

The trait of metal chelation will be discussed in the following section.

However, the question poses: What is the selective advantage of metal hyperaccumulation? Most likely, this special trait offers a defense against pathogen and/or pathogen attack (Freeman et al. 2006Boyd 2007). Nevertheless, fundamental questions concerning mechanisms and properties of hyperaccumulation remain elusive. This knowledge has a broad relevance in general because of accumulation of toxic metals or developments of lacks of essential micronutrients throughout the food chain (idea of biofortification) and for phytoremediation or phytomining processes.

## Heavy metal tolerance and toxicity on cellular level

### Importance of metal speciation on tolerance and toxicity

#### Extracellular metal speciation

Bioavailability of heavy metals in terrestrial ecosystems depends on their physico-chemical form (Blanco et al. 2004), on soil characteristics (Mortvedt 1994) and on growing plant species (Viehweger and Geipel 2010). Another important aspect is bioavailability of essential metals like iron because mechanisms facilitating the uptake of essential metals could provide a gate for non-essential even toxic substances.

A key element of acquisition of nutritional metals is the release of exudates with chelator properties from the roots into the rhizosphere. This complex formation enhances metal solubility and therefore provides a better uptake into the plant. A prominent example is the release of phenolic compounds caused by iron deficiency such as caffeic acid from *Arachis hypogaea* (Römheld and Marschner 1986), flavonoids from *Lupinus albus* (Weisskopf et al. 2006) or flavins from *Beta vulgaris* (Susin et al. 1993) (Cesco et al. 2010). Recent results showed evidence that flavonoids can facilitate heavy metal tolerance in *A. thaliana* (Keilig and Ludwig-Müller 2009). Besides iron chelation, quercetin undergoes a complexation with copper ions (El Hajji et al. 2006Pekal et al. 2011) and uranium (Geipel et al. 2010). An example to underline this issue: Recently a close relation between iron and uranium uptake was shown in *A. halleri*, where iron starvation greatly enhanced uranium uptake (Viehweger and Geipel 2010). The stability constants of U(VI) and Fe(III) bound to Flavinemononucleotide (FMN) are relatively high (log K of 16 for U(VI) and log K of 24 for Fe(III), mononucleotide complexes) (Geipel and Viehweger, unpublished results). Additionally to complex formations, these compounds can exhibit reductive activity towards redox-active metals changing the redox state of metals and therefore their speciation. This complex formation and reduction of non-essential heavy metals has a double edged site: On one hand, it can be used as a defense strategy producing less soluble metal complexes unsuitable for entering the plant. On the other hand, it can stabilize unstable metal redox states and competes with these processes required for acquisition of essential metals.

#### Intracellular metal speciation

If heavy metals enter the cytoplasm they will be bound by an appropriate cellular compound immediately. This avoids the handling of possibly toxic, free cellular metal ions and provides a coordinated involvement in metabolic pathways such as specific incorporation in metalloproteins or detoxification. Ligands for metal ions are mostly low molecular-weight compounds, a comprehensive review was provided by (Haydon and Cobbett 2007).

Well known cellular metal chelators are nicotianamine (NA) and organic acids like citrate (Curie et al. 2009Rauser 1999). NA exhibits very high stability constants for the binding of transition metal cations (for comparison of some stability constants see (Blindauer and Schmid 2010) and is required in *A. thaliana* to maintain iron, zinc and copper homeostasis (Krämer 2010). As it was mentioned in a previous chapter, the concentrations of this chelator are higher in hyperaccumulators than in closely related nonaccumulators (Vacchina et al. 2003). Citrate is the predominant ligand for zinc in leaves of *N. caerulescens* (Salt et al. 1999;Küpper et al. , Küpper et al. Küpper et al. 2000), but other organic acids such as malate are also associated with metal tolerance. (Mihalik et al. 2012) published recently that citrate facilitates uranium translocation from root to shoot interfering iron and zinc transport. This underlines the importance of organic acids for transport and sequestration of metal ions in different plant tissues and cellular compartments like the vacuole (Krämer et al. 2000Ma et al. 2005).

Amino acids like proline are probably involved in metal chelation (Sharma and Dietz, 2006). A key role as nickel chelator has the free amino acid histidine. Nickel increased the histidine content in the xylem of the hyperaccumulator *Alyssum lesbiacum* around 36-fold higher than of the nonaccumulator *Alyssum montanum* (Krämer et al. 1996).

A further important function of metal chelation is the possible enhancement of metal solubility.

Another low molecular-weight chelator is glutathione (reduced form GSH, oxidized form GSSG) which will be discussed in the next chapter. Functionalities of GSH (thiol and carboxylic groups) make it suitable for complex formation with heavy metals (Canovas et al. 2004Frost et al. 2011). Complexation via the thiolate functionality is necessary for the induction of phytochelatines (PCs) (Vatamaniuk et al. 2000). Metal – PC complexes are sequestered into the vacuoles via ABC-type transporters and therefore increase metal tolerance (Mendoza- Cózatl et al. 2010Song et al. 2010). Overexpression of a key enzyme in GSH biosynthesis resulted in higher steady-state levels of GSH and enhanced nickel, cobalt, zinc and to a lesser extent cadmium tolerance in *A. thaliana* (Freeman et al. 2004Freeman and Salt 2007). This increase was proposed to result from GSH mediated reduction of oxidative stress caused by metal exposure (see next chapter).

Metallothioneins (MTs) are sulfur containing proteins inherently being highly flexible in their structure. This flexibility allows different coordination geometries for binding of different metals. Nevertheless, each MT exhibits preferences for a special metal ion due to coordination residues other than cysteine and differences in folding and stability in dependence on the bound metal (Leszczyszyn et al. 2007).

### Impact of heavy metals on the cellular redox environment

Heavy metals interact with the cellular redox environment in different ways:

1. They are able to induce the generation of reactive oxygen species (ROS),
2. Redox-active metals can directly generate ROS via Fenton like reactions and the Haber-Weiss cycle (Stohs and Bagchis 1995Sharma and Dietz 2009)
3. Heavy metal detoxification consumes a major element of cellular redox homeostasis namely glutathione as direct chelator and/or as precursor of phytochelatines.

Because of the outstanding importance of the thiol tripeptide glutathione [γ-Glu-Cys-Gly], this part of the chapter will focus on its interaction with heavy metals in afore mentioned ways.

The tightly regulated cellular glutathione homeostasis (GSH/GSSG, ratios of 100:1 are typical values) is impaired by heavy metal accumulation. This can be one reason for conflicting results regarding the GSH content upon metal exposure. Furthermore, the different points of sampling time should be attended. Copper or cadmium amendment in *Arabidopsis* resulted in only few changes of GSH levels in leaves during the initial 7 days (Collin et al. 2008). (Vandenhove et al. 2006) found altered levels of the glutathione pool depending on the applied uranium concentrations in *Phaseolus vulgaris* after a week of exposure. Concerning the initial phase of metal contact (up to 24 hours), there was no significant change of GSH whereas the amount of GSSG increased upon exposure of moderate uranium concentrations (≤ 10 μM) in cell suspensions of canola (*Brassica napus*) (Viehweger et al. 2011. This indicates clearly its function as redox couple (E_pH7.4_;Schafer and Buettner  = −264 mV at 25°C, Schafer and Buettner Schafer and Buettner 2001) and underlines the consumption of cellular reducing capacity during heavy metal accumulation and causes oxidative stress.

This reductive activity eliminates ROS generated either directly or indirectly by metals. The detoxification of ROS is GSH dependent. Such GSH consuming processes and an excess of ROS induce GSH synthesis (Foyer and Noctor 2005aNoctor et al. 2011). Hence, plant cell are able to cope with moderate imbalances of the glutathione pool. However, higher heavy metal concentrations disrupt these elements of tolerance leading to unspecified reactions of hypersensitive response. Hence, care should be taken when extremely high, environmentally not relevant metal concentrations are applied experimentally.

Another GSH consuming process and thereby impairing the glutathione homeostasis is the synthesis of phytochelatines (PC) (Grill et al. 1985). These heavy metal chelating peptides consist of repetitive γ-glutamylcysteine units and are rapidly synthesized after metal exposure. However, not all heavy metals are capable of inducing PC synthesis such as cobalt or manganese (Grill et al. 1987). A prerequisite is the GSH – metal binding via thiolate coordination (Vatamaniuk et al. 2000). In contrast, heavy metal hyperaccumulators like *N. caerulescens* or *A. halleri* probably do not use GSH or PCs in metal hypertolerance mechanisms (Schat et al. 2002Sun et al. 2007).

Antioxidant defense mechanisms keep the formed ROS at a low level nevertheless heavy metal stress disrupts the equilibrium between ROS generation and detoxification (Sharma and Dietz 2009). Plant cells bear a sophisticated antioxidant network based on non-enzymatic such as glutathione, ascorbate and enzymatic antioxidants like superoxide dismutase (SOD), glutathione reductase (GR) or catalase (CAT). Heavy metal accumulation caused altered capacities of such enzymes depending on plant species. For instance, *Tagets erecta* (Cd accumulator) exhibited decreased levels of SOD, GR and CAT towards cadmium contact whereas *Avena strigosa* (Cd tolerant accumulator) showed increased activities towards the same Cd concentrations (Uraguchi et al. 2006). These few examples illustrate that a strong antioxidant defense system is at least a beneficial trait in heavy metal tolerance (for more examples see Sharma and Dietz, 2009). However, there is no reliable basis for defining mechanistic relationships due to the lack of distinct patterns of enzyme activity. Therefore, the heavy metal, its concentration and the plant species should be carefully taken into account when investigating redox imbalances and oxidative stress induced by heavy metals.

### Relation between toxicity and tolerance

#### Targets of metal toxicity

Symptoms of metal toxicity have been studied in several plant systems and under various conditions. They can be divided in direct targets or indirect metal induced impairments of physiological pathways as it was discussed in previous sections already.

Direct targets of metals are often metalloproteins and metal binding molecules like chlorophyll (Küpper et al. 1996). Metalloproteins contain coordinated transition metals which can be substituted by chemically similar other transition metals (Table 2). A prominent example is the replacement of magnesium by nickel, cobalt or zinc in the enzyme ribulose-1,5-bisphosphate-carboxylase/oxygenase (Wildner and Henkel 1979van Assche and Clijsters 1986) which resulted in loss of enzyme activity. Cadmium interferes with the homeostasis of the essential metals zinc and calcium (reviewed for animal cells in Moulis 2010). A surprising finding was the replacement of iron by uranium in ferritin in the microorganism *Pyrococcus furiosus* (Cvetkovic et al. 2010). The Irving-Williams series provides basic indication for the formation of stable complexes between cations and organic ligands (Irving and Williams 1953). A challenge in the future will be the characterisation of such metalloproteins in-vivo because of few, environmentally relevant metal concentrations which requires sensitive detection systems. Novel technologies like synchrotron X-ray fluorescence microscopy provide a powerful non-destructive technique for quantitative mapping of of transition metal distribution in hydrated biological samples (Fahrni 2007Punshon et al. 2009Donner et al. 2012). An emerging field is the investigation of the redox chemistry driven by some transition metals which can result in dramatic consequences for metabolic pathways.

**Table 2:** **Some examples of metalloproteins which can be modified by metal substitution**

Metal	Native metal	Protein	Reference
Cd, Cu, Fe, Mn, Pb, Zn	Mg	RuBisCo	Reviewed in Van Assche and Clijsters 1990Siedlecka et al. 2001
Cd	Mn	Oxygen evolving complex in photosystem II	Baszynski et al. 1980
U, Al, Pb	Fe	Ferritin	Den Auwer et al. 2005Johnston et al. 1993Cvetkovic et al. 2010
Ni	Zn	Alanyl-tRNA editing hydrolase	Cvetkovic et al. 2010
Zn	Ca	Endonuclease	McCabe et al. 1992
Co	Zn	ATP sulfurylase	Gavel et al. 1998
Pb, Cd	Ca	Calmodulin	Habermann et al. 1983Richardt et al. 1986

It should be noted that not all examples were determined in plants.

Beside afore mentioned interactions between essential and non-essential heavy metals, such competitions can also occur between micronutrients (Foy et al. 1978). Known examples are interactions between iron and copper (Harris 1994) or iron and zinc (House 1999). These influences are mostly negative nevertheless there can be positive growth parameters in the case of antagonistic responses according to the classification done by Symeonidis and Karataglis (1992). A comprehensive review addressing such interactions was written by (Krupa et al. 2002).

These few examples underlines that heavy metal toxicity is partly due to the impairment of the tightly regulated homeostasis of essential metals.

#### Mechanisms of tolerance

As it mentioned earlier, plant cells possess a complex network for coping with heavy metals and some reactions have been touched upon in this article.

Generally speaking, the key elements of basal tolerance are sequestration and efflux (Clemens 2001). These processes resulted in the removal of toxic ions from sensitive cellular loci. Thereby transport is facilitated by metal chelators in most cases (see previous section) and requires efficient transport systems.

In addition to previously discussed cellular metal ligands, metallochaperones play an important role in facilitation of essential metals to reach the physiological destination in distinct cellular compartments without inflicting damage. A comprehensive review concerning copper metallochaperones was published by (Robinson and Winge 2010) recently. Another cellular strategy for the insertion of the correct metal cofactor into metalloproteins (metallation) is compartmentalization. Therefore distinct metals are stored at different subcellular compartments and controlled delivered to their final destinations via specific transport systems (Tottey et al. 2008).

Plant cells possess a variety of transport systems including the already mentioned heavy metal ATPases (HMAs), Nramps (natural resistance associated macrophage proteins), the cation diffusion facilitator (CDF) family, the ZIP (ZRT, IRT-like proteins) family, ABC transporters (ATP-binding cassette), cation antiporters and other putative transition metal transporters. A more detailed overview is provided by reviews written by Guerinot (2000), Hall and Williams (2003), Krämer et al. (2007) or (Puig and Penarrubia 2009) and references therein. Although they play a crucial role in maintenance the homeostasis of essential metal micronutrients they are involved in detoxification of non-essential or excess metal. Their overexpression and higher cellular density as special trait in metal hyperaccumulators was discussed in a previous chapter. Various transport systems can act as a gate for various metals because of chemical similarities of metal ions or their complexes. A prominent example for this is the iron-regulated transporter (IRT1) at the plasma membrane of roots of *A. thaliana* which exhibits a broad substrate range (Korshunova et al. 1999).

As it was mentioned before, metal sequestration in distinct cellular compartments plays a pivotal role in metal tolerance and supplement with essential metals. For this purpose cells provide a coordinated set of transport systems in each cellular membrane. An important metal sink in metal tolerant plants is the vacuole. A prominent example is the transport of metal-phytochelatin complexes into the vacuole by an unknown ABC transporter or by cation/proton exchanger (CAX) (Schneider et al. 2009). However, recent studies suggested that the phytochelatin-cadmium complex formation plays a more important role in detoxification than vacuole sequestration (Wojas et al. 2010).

## Signals generated by heavy metal exposure

As it was discussed in previous sections, heavy metals interact with metabolic pathways and therefore are able to generate signals. Thereby, the similarity of chemical properties of different metals plays a pivotal role especially between essential and non-essential metals. However, such interaction does not inevitably have a negative impact for the plant at all points. For instance, *Arabidopsis* or carrot plants exhibited an obvious better root elongation when growing with uranium amendment (Straczek et al. 2009Misson et al. 2009Viehweger and Geipel 2010) probably due to the excretion of phenolic compounds into the rhizosphere which can stimulate root elongation (Wang 1991). This example shows as well the importance of investigations concerning whole networks of metal metabolism. Recent experiments suggested a facilitated iron acquisition in *A. halleri* growing on a former uranium mining dump which provided very low soluble iron minerals (Viehweger and Geipel 2010, unpublished results). Table 3 provides an insight in the complex signaling network induced by various environmental stress conditions.

**Table 3:** **Overview of some heavy metal triggered signals in comparison to other environmental stresses**

Heavy metal	Signal	Other stress conditions	Cellular responses	References
Copper	Calcium fluxes	Cold, drought, salinity	Phosphoprotein cascades, 2^nd^ signalling molecules	Nielson et al. Nielsen et al. 2003reviewed by Sanders et al. 1999Knight 2000
Cadmium, chromium	Mitogen activated protein kinase (MAPK) pathways	Osmotic stress, pathogen contact	Activation of transcription factors and stress-responsive genes	Liu et al. 2010Ding et al. 2009, reviewed in Xiong et al. 2002
Iron	pH shifts	Pathogen contact	Induction of secondary metabolism	;Marschner and Römheld 1983Viehweger et al. 2006
Cobalt, zinc	Plant hormones like abscisic acid or ethylene	Cold, drought, salinity	Calcium signalling, guard cell regulation (water balance)	Zengin 2006reviewed in Zhu 2002
Cadmium, copper	Jasmonic acid	Pathogen contact, sugar, drought, salinity	Defence/stress response, development, induction of secondary metabolism	Agrawal et al. 2003Chen et al. 2006Maksymiec 2011reviewed in Howe and Schilmiller 2002Turner et al. 2002
Redox-active metals like iron, copper; almost all heavy metals at higher concentrations	Reactive oxygen species	Pathogen contact, cold, drought, salinity, high light intensity	Phosphoprotein cascades, activation of transcription factors and stress-responsive genes, activation of antioxidative defence	Reviewed in Foyer and Noctor 2005aAhmad et al. 2008

It is not comprehensive but gives an insight in the complex cellular signalling network and the resultant metabolic reactions.

### Signal perception

Generally, a signal transduction pathway starts with the signal perception in the case of heavy metals with its recognition by the cell. Up to date, much less knowledge is available about a primary recognition for instance by a receptor. It is likely that the metal is mainly perceived by plasma membrane proteins responsible for metal acquisition like reductases and transporters. However, other sensors can be expected resulting from physical alterations in cellular structures induced by metal exposure. If the metal is recognised by cells the further cellular signal transduction will use elements of common signalling pathways like calcium fluxes.

### Cellular secondary signal molecules

The most prominent secondary messenger is calcium (reviewed e.g. in Xiong and Zhu 2002). Treatments of the brown algae *Fucus serratus* with different copper concentrations inhibited or induced cytoplasmic calcium fluxes (Nielsen et al. 2003). These alternate fluxes might initiate calcium dependent protein kinases and therefore couple this universal inorganic signal to specific protein phosphorylation cascades like MAP kinase activities (Yeh et al. 2007).

Another inorganic signal is the proton which also enables fast concentration shifts (but not so fast like calcium). Proton fluxes can establish so-called “pH-signatures” in the cytoplasm (Roos et al. 1998) and thus creating specific micro-areas with for example enhanced metal solubility. Iron deficiency causes a proton efflux driven by a P-type proton ATPase (AHA7) acidifying the rhizosphere (Santi and Schmidt 2009) which induces a pH-shift in the near neutral cytoplasm. However, nothing is known about a possible impact on cellular signaling.

As it was mentioned in chapter 3.2 heavy metals are able to elicit the production of ROS. Beside their activity as aggressively reacting oxidants towards cellular macromolecules they can act as signal transduction molecules which will be discussed in Section Redox signaling induced by heavy metals.

### Stress signal transduction by plant hormones

During heavy metal exposure, plants produce increased amounts of hormones such as abscisic acid or ethylene (Zengin 2006Maksymiec 2011). Additionally, the involvement of jasmonic acid in the early response to cadmium contact in *Phaseolus coccineus* was described recently by Maksymiec (2011). Hence, it seems to be obviously, that elements of octadecanoid pathway interacts in metal induced signalling and even act in potential defence reactions. This pathway is part of the oxidative/redox system of plants which was discussed in a previous section already. It underlines the tight regulation of metal homeostasis in a widespread network consisting of various elements.

### Signaling by transcription factors

A well investigated example is the function of transcription factors (TFs) during iron starvation in plants. A detailed review concerning this was published recently (Hindt and Guerinot 2012). Early results obtained by Brown and Chaney (1971) and Brown and Ambler (1974) suggested a pivotal role of a basic helix-loop-helix (bHLH) transcription factor (named FER) in iron deficiency responses using an iron inefficient tomato mutant (*Lycopersicon esculentum*). FER encodes bHLH which controls the expression of genes with key functions in iron acquisition such as the iron-regulated transporter (IRT) and the ferric oxidase reductase (FRO) orthologs in tomato (Ling et al. 2002). The Arabidopsis FER ortholog is FER-like iron deficiency-induced transcription factor (FIT) (Colangelo and Guerinot 2004Jakoby et al. 2004Yuan et al. 2005). FIT form heterodimers with two other bHLH proteins (Yuan et al. 2008) and is iron responsive. Interestingly, two TFs of the bHLH family in *Arabidopsis* are involved in the iron deficiency induced synthesis and excretion of riboflavin when heterologous expressed in tobacco and sunflower (Vorwieger et al. 2007). This example underlines that TFs are essential components in the regulatory pathway connecting perception of iron deficiency and cellular reactions. Recent results provided evidence for another set of transcription factors called POPEYE network (Long et al. 2010). POPEYE (PYE) is a recently identified bHLH transcription factor, the other player is the putative E3-ubiquitin ligase called BRUTUS (BRS). Both are induced by iron deficiency in roots. The importance of this network was revealed using the pye-1 mutant which suffered from chlorosis and poor root growth under iron starvation.

Nevertheless, TFs are although induced by other heavy metals. These TFs can be involved in other cellular processes and therefore may interfere with them. For instance, ethylene-responsive element-binding factor (ERF) gene expression was detected *A. thaliana* and *A. halleri* after cadmium treatment (Weber et al. 2006). Dehydration-responsive element-binding protein (DREB) TFs can be either up- or down-regulated by heavy metals like cadmium and copper (Ogawa et al. 2009;Ban et al. 2011). Genes encoding TFs belonging to bZIP, Myb, and zinc-finger protein families are up-regulated by cadmium exposure found by (Fusco et al. 2005), recently. The regulation of TFs plays an important role in maintaining metal-hyperaccumulating abilities. (van de Mortel et al. 2006) revealed that 131 TFs showed a more than 5-fold higher expression in *T. caerulescens* compared to *A. thaliana* under zinc sufficient conditions. As the terms of the discussed TFs suggested they were discovered as cellular responses after various stresses. This strengthens the idea of common response mechanisms different against biotic and abiotic threats.

### Redox signaling induced by heavy metals

As it was discussed in a previous chapter heavy metal exposure can cause imbalances in the cellular redox homeostasis either by being itself redox-active or by replacing other metal ions (sometimes even redox-active ions) in biomolecules. A recently discussed issue in animal cells is the zinc coordination with sulphur donors of cysteine resulting in a redox switch with reversible oxidoreduction of the sulfur donor linked to zinc association and dissociation (Maret 2012). This phenomenon might be adopted in plant cells too and bridges the gap between coordination chemistry of metal ions in biomolecules and their resultant function.

A fundamental role in signaling is fulfilled by the generated reactive oxygen species (ROS) which can activate mitogen-activated protein kinase (MAPK) cascades in a plant species and metal dependent manner (Gupta and Luan 2003Rentel et al. 2004). As an example, (Liu et al. 2010) demonstrated an activation of MPK3 and MPK6 via accumulation of ROS in *Arabidopsis*. These cascades end up by phosphorylation of transcription factors (see Cellular secondary signal molecules) interacting with gene promoters and thus inducing gene expressions. It should be noted that there are differences between metal-sensitive and metal-tolerant plants: ROS-MAPK signals cause several cellular damages like interruption of hormonal signalling, programmed cell death in metal-sensitive plants. In contrast, metal-tolerant plants are able to accumulate repair proteins such as chitinases and heat shock proteins (HSP). A more detailed overview provides a recently published review by Lin and Aarts (2012). However, it should keep in mind that ROS is produced as a response to myriad kinds of stresses and therefore could interfere with other cellular reactions. Generation of ROS after heavy metal contact is mostly an unspecific reaction due to abnormal high metal concentrations. It is a common phenomenon that excessive biotic or abiotic threats elicit nondirectional responses.

Other important elements are the soluble redox couples like glutathione or NAD(H), NADP(H) which provide a buffering system in the cytoplasm (Noctor 2006). Local perturbation of this system is likely a step in signal transduction processes inducing e.g. enzyme or hormone activity. These processes occur in all cellular compartments reflecting their physiological importance. Comprehensive reviews concerning this sophisticated network can be found in Foyer and Noctor ([Bibr CR33][Bibr CR34]), Grene (2002) and (Ahmad et al. 2008).

## Conclusion and further perspective

Almost all plants exhibit a basal metal tolerance when facing heavy metals. Some species are even capable of hyperaccumulation running different tolerance reactions compared with nonaccumulating plants. However, general tolerance mechanisms are based on exclusion, chelation and sequestration processes (Figure 1).

Attention should be paid on signal transduction pathways induced by metals because they use common signal elements which can be also elicited by other environmental stresses. The critical evaluation of triggered signals and their responses is mandatory for understanding metal homeostasis. The challenge in the future will be the investigation of multiple stress factors as it occurs under real environmental conditions.

Special focus should put on low, environmentally relevant heavy metal concentrations. Therefore, the further development of sensitive detection methods and the combination of different approaches are necessary. These tools enable for instance new insights in the metalloproteome and its interactions (“metallomics”). Further knowledge about metal tolerance in plants is mandatory for several purposes:

1. Predictions about health risk which is caused by metal accumulation in crop plants failing visible symptoms of phytotoxicity.
2. Generation of genetically engineered plants having an enhanced accumulation of metals valuable for nutritional purposes (biofortification).
3. Clean up of metal contaminated soils (phytoremediation) and mining of rare metals which are accumulated in plant tissues (phytomining).

## Authors’ original submitted files for images

Below are the links to the authors’ original submitted files for images. Authors’ original file for figure 1
