# Supplementary figures and images for: How plants cope with heavy metals
Source: Bot Stud. 2014 Mar 20;55:35. doi: 10.1186/1999-3110-55-35 (PMC5432744; doi:10.1186/1999-3110-55-35)

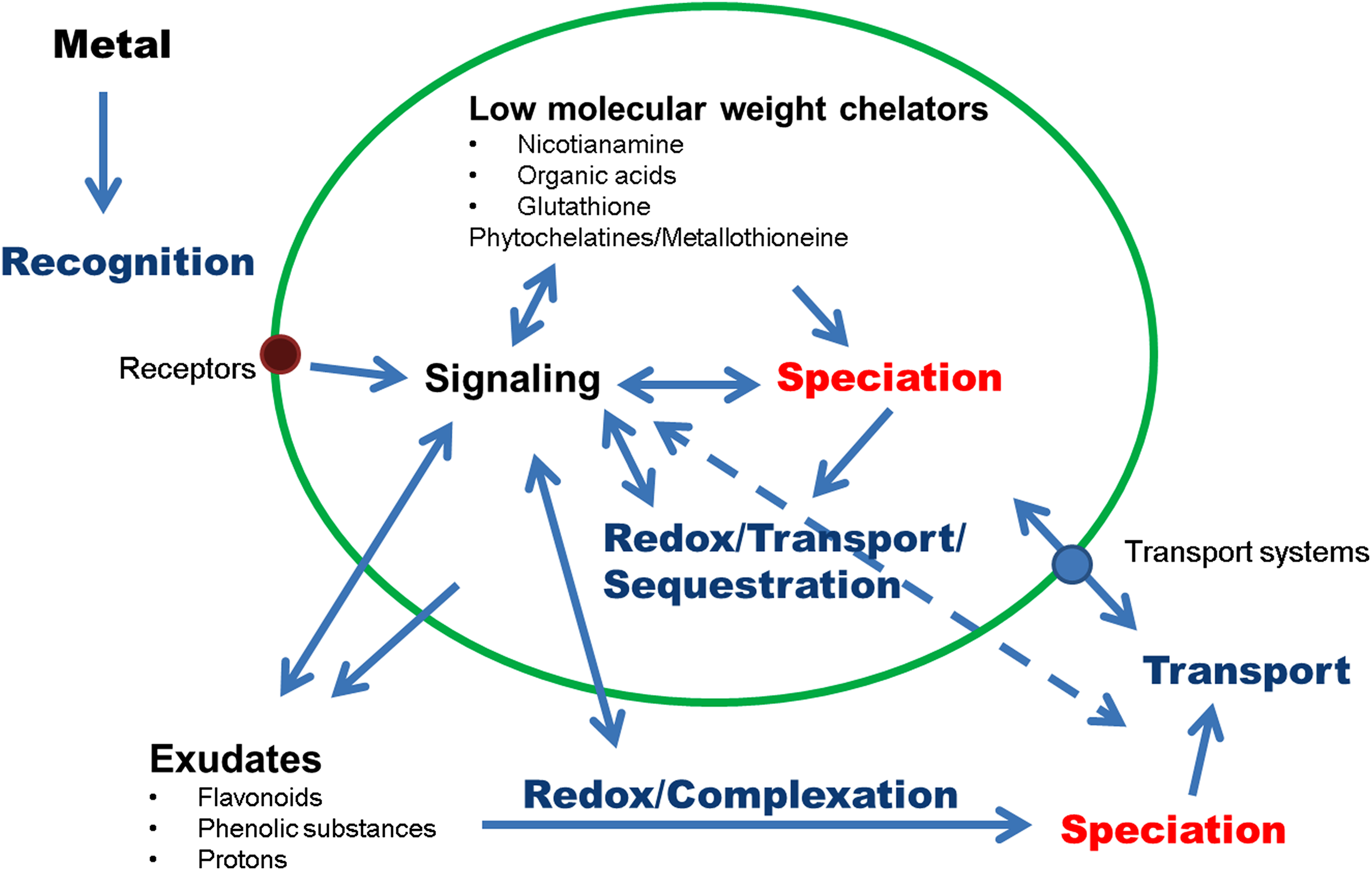

Supplement: Supplementary file 1 — Authors’ original file for figure 1 [file 40529_2012_81_MOESM1_ESM.tiff]
